# Neurosyphilis as a cause for neuropsychiatric symptoms: a case report

**DOI:** 10.1192/j.eurpsy.2022.1684

**Published:** 2022-09-01

**Authors:** I. Romero Gerechter, M.D.C. Molina Liétor

**Affiliations:** Hospital Universitario Príncipe de Asturias, Psiquiatría, Alcalá de Henares, Spain

**Keywords:** Syphilis, mania, neuroshyphilis, old people psychiatry

## Abstract

**Introduction:**

Syphilis is a sexually transmitted infection which in its late phase can cause all kinds of neuropsychiatric symptoms. A case report of a 79-year-old male with a manic episode probably due to lues is presented.

**Objectives:**

A case of a patient with neurosyphilis is presented followed by a theoretical review on the topic.

**Methods:**

A case is presented with a bibliographic review.

**Results:**

A 79-year-old male was hospitalized with symptoms of disorientation, inattention, and difficulty for abstract thought. His speech was verbose, incoherent with megalomaniac ideas. He presented affective symptoms such as hyperthymia, emotional lability and intermittent crying. He also had nomination problems and recent memory mistakes. He also suffered from insomnia. He presented as his medical history HIV infection under control and syphilis treated in December 2020 with a negative RPR test in June 2021. During his hospitalization he was treated with increasing doses of olanzapine and valproic acid. Irritability improved with this treatment.

**Conclusions:**

Neurosyphilis may be presented as any kind of neuropsychiatric disorder. Laboratory tests are required as there is no conclusive imaging test. Penicillin and symptomatic management are the proper treatment. Neuropsychiatric disorders in elderly population must consider infectious diseases and previous pathologies as differential diagnosis.

**Disclosure:**

No significant relationships.

